# Evaluation of the position of the posterior superior alveolar artery in relation to the maxillary sinus using the Cone-Beam computed tomography scans

**DOI:** 10.4317/jced.53213

**Published:** 2017-03-01

**Authors:** Mohammad-Taghi Chitsazi, Adileh Shirmohammadi, Masoumeh Faramarzi, Farzad Esmaieli, Shadi Chitsazi

**Affiliations:** 1 Professor, Department of Periodontics, Tabriz Faculty of Dentistry; 2 Associate Professor, Department of Periodontics, Tabriz Faculty of Dentistry; 3 Associate Professor, Department of Maxillofacial Radiology, Tabriz Faculty of Dentistry; 4 Dentist, Private Practice

## Abstract

**Background:**

The aim of the present study was to evaluate the diameter, relationship and position of the posterior superior alveolar artery and its relationship with the alveolar ridge, the medial wall of the maxillary sinus, the prevalence of pathologic conditions and the maxillary sinus septa on CBCT images.

**Material and Methods:**

A total of 200 CBCT images (400 maxillary sinuses) of patients over 20 years of age were evaluated. The distances between the lower border of the artery and the alveolar crest and between the artery and the medial wall of the sinus and the diameter of the artery were measured. The position of the artery, the presence of pathologic conditions and septa were recorded in the posterior region in: a) males edentulous in the posterior region; b) males having teeth in the posterior region; c) females edentulous in the posterior region; and d) females having teeth in the posterior region.

**Results:**

The mean distance between the artery and the alveolar crest, irrespective of groupings, was 16.17±1.63 mm, with significant differences between the groups (*P*<0.05). The mean distance between the artery and the medial wall of the sinus was 11.65±1.21 mm, with no significant differences between the groups (*P*=0.796). The mean diameter of the canal was 1.37±0.44 mm, with no significant differences between the 4 groups (*P*=0.570). The position of the artery was intraosseous in 73.2%, beneath the sinus membrane in 21.7% and external to the lateral wall of the sinus in 4.9% of the cases. The overall prevalence rates of pathologic conditions and septa in the maxillary sinus were 45.7% and 26%, respectively.

**Conclusions:**

CBCT technique is useful for such evaluations and for possible variations in maxillary sinuses and presence of septa and pathologic entities in maxillary sinuses.

** Key words:**Maxillary sinus, maxillary artery, Cone-Beam computed tomography.

## Introduction

A common limitation in relation to the placement of dental implants is a lack of adequate bone in the edentulous area (1). One of the areas which might undergo alveolar bone atrophy and exhibit poor quality of bone is the posterior maxilla. The maxillary sinus is the paranasal sinus that affectss most the work of the dentists and the maxillofacial surgeon when treatment requires bone grafting in this area. The presence of the maxillary sinus complicates the problem of inadequate bone because there is an increase in the size of the maxillary sinus over time, and the proximity of the floor of the sinus to the alveolar ridge results in a decrease in bone height, making it impossible to place dental implants (2). To overcome such a problem various techniques have been suggested for the osseous reconstruction of the sinus floor and different materials have been used as grafts in this region. In fact, the majority of the techniques used are safe and at the same time predictable (3). However, serious complications might arise due to the improper implementation of the techniques and treatment procedures (4). All the surgical procedures in the posterior maxilla require a proper recognition of the anatomic details of maxillary sinuses and the possible anatomic variations in this area (5). The blood supply of the maxillary sinus is provided by the branches of the maxillary artery, i.e. the posterior superior artery (PSAA) and the infraorbital artery (6). These arteries supply blood to the mucous membrane of the sinus, the periosteum and the antero-lateral wall of the maxillary sinus (7). Based on a number of previous studies, these arterial branches are always found in the sinus wall; however, their location, diameter and route might be different. On the other hand, their distance from the alveolar ridge depends on the atrophy of the maxillary bone (8). Misch and Judy (9) classified the edentulous alveolar ridge on the basis of bone height as ≤10 mm or >10 mm. Khojastehpour *et al.* (10) evaluated the diameter, location and frequency of the appearance in preoperative cone-beam computed tomography (CBCT) scans and reported that the distance between the artery and the medial sinus wall, as well as the diameter of the artery, were greater in patients with an alveolar bone height of ≤10 mm compared to those with a bone height of >10 mm. The distance from the artery to the medial sinus wall and the diameter of the artery were positively correlated with the number of missing teeth. It was also found that the diameter of the PSAA increased with increasing age. However, Danesh-Sani *et al.* (11)showed no significant correlation between age and the size of the PSAA.

The branches of the maxillary artery should be taken into consideration during sinus lift. During sinus augmentation procedures, these branches are at a risk of hemorrhage due to injuries to the arteries in the lateral wall of maxillary sinuses (12). Any imaging technique should provide the operator with information about this region as far as possible. CBCT might be recommended as a technique with a low radiation dose, compared to conventional medical tomographic scans, for imaging of the dentate region of the maxillofacial area (12). The radiation doses of medical tomographic scans are 1.5 to 12.3 times of those of CBCT. Therefore, this imaging technique is mostly used for the evaluation of the alveolar ridge and maxillary sinus in patients receiving dental implants (13). Most previous studies have evaluated the mean diameter of PSAA, path of this artery, location and relationship of this artery to the alveolar ridge (5,8,12). But these studies have not investigated the location of PSAA based on sex and dental status.

The aim of the present study was to evaluate the relationship and location of posterior superior alveolar artery in relation to the alveolar ridge and the medial wall of maxillary sinus, the prevalence of pathologic conditions and septa in maxillary sinuses and comparison of location of artery in terms of the type of edentulism and sex, using CBCT imaging technique.

## Material and Methods

In this retrospective study, a total of 200 CBCT images (400 maxillary sinuses) were evaluated in patients over 20 years of age, who referred to a private clinic. A written informed consent form, which is routinely obtained from each patient prior to imaging in our clinic, also included a clause for the use of images in this research.

All the scans had a high quality to be included in this research and were taken using a scanner. CBCT images of maxillary sinuses that were damaged by tumors or severe traumas were excluded from this study. The images had been taken with a Planmeca Promax 3D CBCT unit (Helsinki, Finland) at kVp=84 and mA=16, using 1-mmm cross-sections. For intra-examiner calibration and determination of the reliability and reproducibility of the measurements, the images were re-evaluated by the same observer 2 weeks after the initial evaluation.

-Data collection 

The scans were evaluated in the coronal dimension by a maxillofacial radiologist in three cross-sections: First in the axial cross-section in which the artery was observed for the first time; the second cross-section, after which the artery was not seen; and the third cross-section between these two cross-sections. The position of the artery was divided into: a) intraosseous; b) submembranous; c) on the external cortex of the lateral wall of the sinus. The male and female subjects were divided into 4 groups as follows: 1) males edentulous in the posterior region (n=42); 2) males dentate in the posterior region (n=32); 3) females edentulous in the posterior region (n=37); and 4) females dentate in the posterior region (n=31). The diameters of the artery in the three groups were as follows: a) less than 1 mm; b) 1-2 mm; c) over 2 mm. The distance between the inferior border of the artery and the alveolar crest and the distance between the artery and the medial wall of the sinus were determined.

The presence of pathologic entities, including thickening of the membrane, chronic sinusitis and cysts, and the presence of sinus septa measuring at least 2 mm, were recorded. In addition, the canal diameter was recorded in relation to age, gender and location. To determine reliability, the scans were randomly re-evaluated after a month by the radiologist.

-Statistical analyses

SPSS 23 was used for statistical analyses. Kolmogorov-Smirnov test was used for the evaluation of normal distribution of data. Intra-class correlation coefficient (ICC) was used to determine agreement between the two observations.

Means and standard deviations were used to show the distance between the inferior border of the artery and the alveolar crest and also the distance between the artery and the medial wall of the sinus. One-way ANOVA was used to evaluate differences between male and female subjects with and without teeth in the posterior region. In addition, the means and standard deviation of the diameter of arteries were calculated and divided into three groups based on percentages: a) less than 1 mm; b) 1-2 mm; and c) over 2 mm. One-way ANOVA was used to evaluate differences between males and females with and without teeth in the posterior region and the percentages of the location of the artery in terms of intraosseous, submembranous or on the external cortex of the lateral wall of the sinus were separately calculated. The presence of pathologic conditions irrespective of the etiology, including an increase in the thickness of the sinus mucosa to more than 2 mm and the percentage of the presence of sinus septa (at least 2 mm), was determined. The means and standard deviations of canal diameter were determined in male and female subjects. One-way ANOVA was used to evaluate differences between males and females with and without teeth in the posterior region. In addition, Pearson’s correlation coefficient was used to determine the correlation between age and the canal diameter.

## Results

A total of 400 left and right maxillary sinuses were evaluated. The posterior superior alveolar artery canal was observed in 71% of the sinuses, with 73.2% of the arteries being intraosseous, 21.7% under the sinus membrane and 4.9% external to the lateral wall of the sinus. Location of PSAA is described in  Table 1.

**Table 1:**
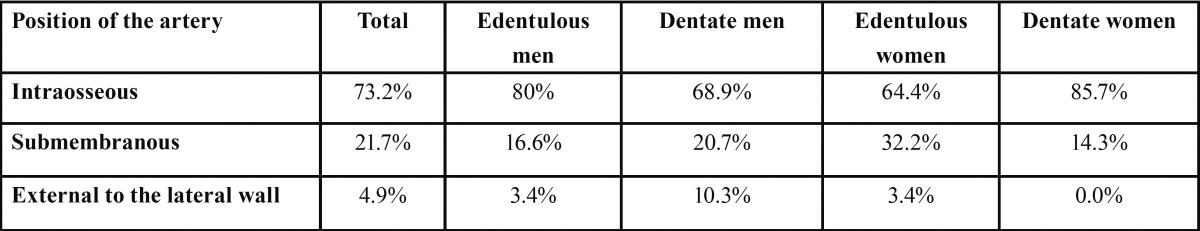
Percentages of the position of the artery in the 4 study groups.

 Table 2 presents classification of the canal diameter based on percentages in males and females, which were divided into 3 groups: a) less than 1 mm; b) 1-2 mm; and c) over 2 mm.

**Table 2:**
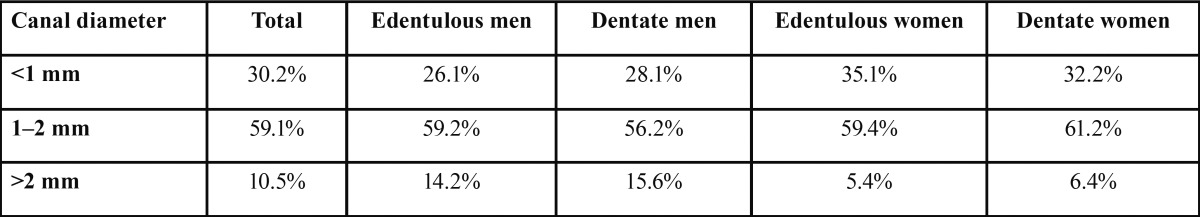
Percentages of canal diameters separately in the 3 pre-defined groups.

In general, the canal diameters were less than 1 mm in 30.2%, 1-2 mm in 59.1% and more than 2 mm in 10.5% of the cases. Range of diameter of the canal was 0.7- 2.7 mm in this research.

The means in males and females with and without teeth in the posterior region are described in  Table 3.

**Table 3:**
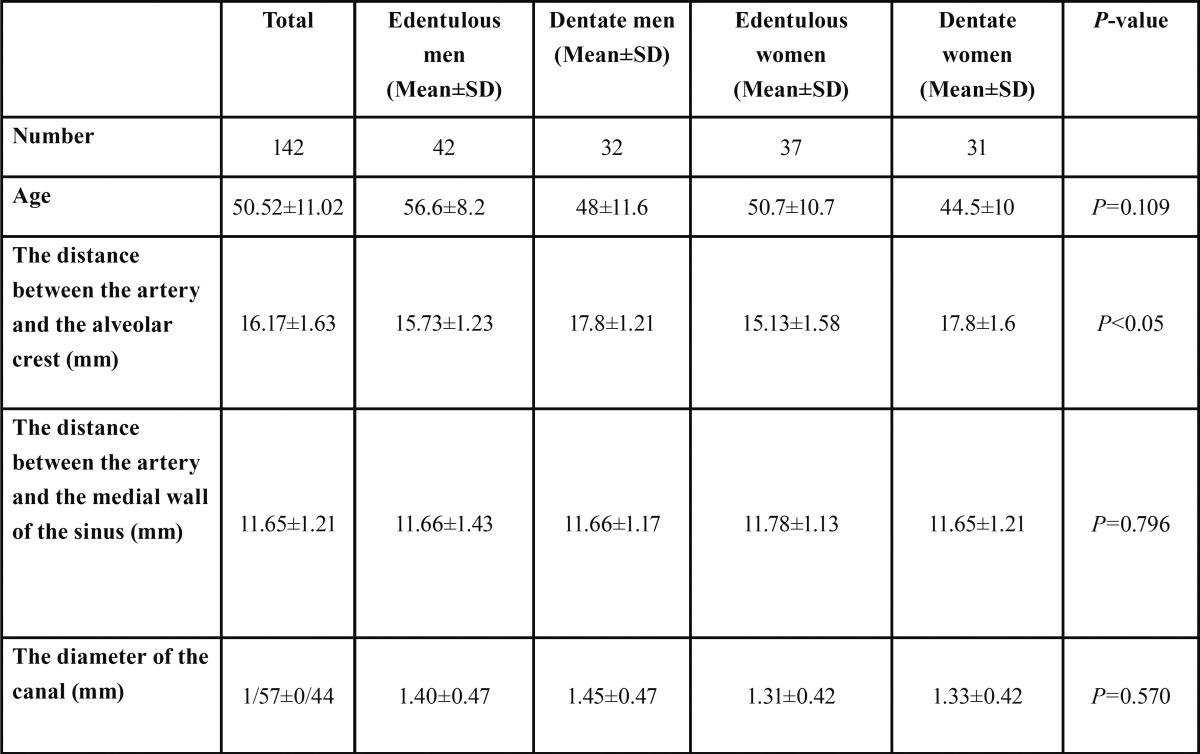
The position of the posterior superior alveolar artery in the 4 study groups and the diameter of canal.

The mean distance between the artery and the alveolar crest, irrespective of the groups, was 16.17±1.63 mm. The distances in edentulous males, dentate males, edentulous females and dentate females were presented in  Table 3. Range of distance between artery to the alveolar crest and artery to medial wall of sinus was 14.3-22.3 mm and 9.2-15.3 mm, respectively.

The overall prevalence of pathologic conditions in the study groups is presented in  Table 4. In addition, the overall prevalence of septa within the maxillary sinus was 26%, with 28%, 25%, 27% and 25% in edentulous males, dentate males, edentulous females and dentate females, respectively ( Table 4).

**Table 4:**
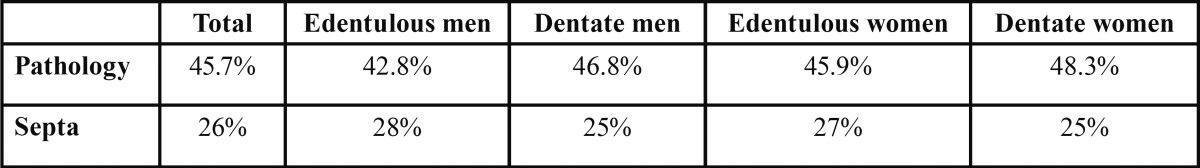
Prevalence of pathologic conditions and septa within the maxillary sinuses in the 4 study groups.

## Discussion

Surgical procedures in the posterior maxilla to place dental implants require full knowledge about the anatomic details and possible variations in the anatomy of maxillary sinuses (14). In the present study, the posterior superior alveolar artery canal was observed in 71% of maxillary sinuses evaluated, consistent with previous works (5,15). Location of artery was intraosseous in the majority of cases (73.2%). Güncü *et al.* (2011), Ilgüy *et al.* (2013), Elian *et al.* (2005) and Kim *et al.* (2011) reported observation of the canal in 89.3%, 64.5%, 52.9% and 52% of the cases, respectively (5,12,16,17). Although the results of the present study, similar to those of other studies, are different, an anatomical study by Kqiku *et al.* (2013) reported observation of the canal in 100% of the cases (6), possibly confirming the hypothesis that the canal is present in all the samples and the differences are possibly due to the use of different techniques and devices, the small diameter of the canal and a lack of accurate observations.

In addition, in the present study, the canal diameters were less than 1 mm, 1-2 mm and more than 2 mm in 30.2%, 59.1% and 10.5% of the cases, respectively, with an overall mean diameter of 1.37±0.44 mm, irrespective of the groups. Güncü *et al.* (5) and Kim *et al.* (17) reported mean canal diameters of 1.3±0.5 and 1.52±0.47 mm, consistent with the results of the present study. However, Ilgüy *et al.* (12) reported a mean canal diameter of 0.94±0.26 mm, which is less than that in the present study. On the other hand, Solar *et al.* (18) reported a mean canal diameter of 1.6 mm in an anatomical study. Given the results of different studies and based on the results of anatomical studies, it is possible that discrepancies in the results of these studies might be attributed to the use of different radiographic devices. On the other hands, this can explain the results reported by Abboud *et al.* (19), who reported the results of CBCT technique to be less than those of the CT technique. In the present study, the canal diameter was less than 1 mm in 30.2% of the cases, consistent with the results of a study by Güncü *et al.* (5) (36.1%) and Mardinger *et al.* (20) (26%); however, Ilgüy *et al.* (12) and Kim *et al.* (17) reported higher (68.9%) and lower (13.9%) rates, respectively. In the present study, the canal diameter was 1-2 mm in 59.1% and more than 2 mm in 10.5% of the cases, with no significant differences between the groups under study (*P*=0.570). Consistent with the present study, Beretta *et al.* (14) and Mardinger *et al.* (20) did not report a relationship between canal diameter and gender. It is obvious that the canal diameter has a direct relationship with the amount and severity of hemorrhage and it is necessary for the clinician to pay special attention to the canal diameter in the CBCT technique in order to be able to predict possible complications and take measures before the surgical procedure. In the present study, the mean distance between the posterior superior alveolar artery and the alveolar ridge was 16.17±1.63 and based on  Table 3, the differences in the means between the groups were significant. According to  Table 3, the mean distance between the artery and the alveolar crest in subjects with teeth in the posterior region (males and females) was almost 2 mm different from that in the subjects edentulous in the posterior region (males and females).

Based on the results of a study by Van der Weijden *et al.* (21), the mean decrease in the alveolar crest height was 1.67, which is justifiable. The results of the present study are consistent with those of studies by Güncü *et al.*, Watanabe *et al.*, Kim *et al.* and Mardinger *et al.*, who reported distances of 18.9, 16.4, 16.9 and 18 mm between the alveolar crest and artery (5,15,17,20); Ilgüy *et al.* (12) reported this distance to be 17 mm in females and 16.79 mm in males. On the other hand, Kqiku *et al.* (6) reported this distance to be 14.66-17.72 mm in dentate jaws in an anatomical study. The results of the present study are within the range reported by Kqiku *et al.* (6). In the present study and similar studies, the reference point was the inferior border of the alveolar ridge but as a matter of fact the issue of alveolar bone resorption has not been settled and it is certain that such resorption is different in different samples. Therefore, it is wise for the clinician to consider resorbed ridges for further evaluations. However, on the other hand, since CBCT underestimates distances (22), the clinician can trust this measurement by considering the amount of ridge resorption and if the canal is visible, the clinician should be fully prepared to manage the possible hemorrhage from this artery. However, lack of observation of this canal is a possible indication that the canal diameter is less than 0.5 mm.

In the present study, the overall mean distance between the artery and the medial wall of the sinus, irrespective of the groups, was 11.65±1.21 mm, with no significant differences between the groups. Ilgüy *et al.* (12) reported this distance to be 13.27±2.82 and 14.03±2.44 mm in females and males, respectively; Güncü *et al.* (5) reported this distance to be 11±3.8 mm, consistent with the results of the present study.

The prevalence of pathologic conditions was 45.7% in the present study. Ilgüy *et al.* (12) and Güncü *et al.* (5) reported prevalence rates of 57.4% and 24.8%, respectively, for pathologic conditions. In the present study, an increase in the mucosal thickness of the sinus was considered a pathologic condition, irrespective of its etiology (21). Although the results of the present study are, to some extent, similar to those reported by Ilgüy *et al.* (12), they are very different from those reported by Güncü *et al.* (5). Ilgüy *et al.* (12), similar to the present study, considered an increase in the thickness of sinus mucosa as a pathologic entity but did not score the severity of the pathology. On the other hand, Güncü *et al.* (5) did not report any criteria for the pathologic condition of the mucous membrane; therefore, it is not possible to explain such a discrepancy. On the other hand, the prevalence of septa in the maxillary sinus, irrespective of its location and extent, was 26%, which was reported to be 55.2% in the study by Misch *et al.* (9), with a significant difference from the present study. However, Güncü and Krennmair reported prevalence rates of 16% and 16.1%, respectively (5,23), which is slightly different from the results of the present study. In this context, Kim *et al.* (17) and Pommer *et al.* (24) reported prevalence rates of 26.5% and 28.4%, respectively, consistent with the results of the present study. Ilgüy *et al.* (12) compared their results with those of studies by Orhan *et al.* (58%), Neugebauer *et al.* (47%) and Lana (44.4%) (25-27) and believed that the consistency between the results might be attributed to the use of CBCT, also believing that a decrease in the observation rate of sinus septa in these studies might be due to the use of CT in these studies. However, Abbound *et al.* (2013) showed in a comparative study that CT was more accurate than CBCT (19). An anatomical study by Gosau *et al.* (28) showed a prevalence rate of 27% for the septa, possibly confirming the results of the present study. It appears discrepancies between the reports on septa might be attributed to the number of samples, the radiographic techniques used, accuracy of observations, definition in relation to the size and height of the septa and the presence or absence of teeth in the area in question. The limitation of present study was the lack of separation of pathological lesions. Therefore, further studies with histological evaluations are recommended.

## Conclusions

The present study recommends the use of the CBCT technique for the evaluation of the position and status of the posterior superior alveolar artery in order to prevent possible complications during implant placement.
